# Enhanced Anxiety Observed in Cocaine Withdrawn Rats Is Associated with Altered Reactivity of the Dorsomedial Prefrontal Cortex

**DOI:** 10.1371/journal.pone.0043535

**Published:** 2012-08-16

**Authors:** Cynthia El Hage, Virginie Rappeneau, Adeline Etievant, Anne-Laure Morel, Hélène Scarna, Luc Zimmer, Anne Bérod

**Affiliations:** 1 INSERM, U1028, Lyon Neuroscience Research Center, Physiopathology of the neuronal network responsible for the sleep-waking cycle Team, Lyon, France; 2 CNRS, UMR5292, Lyon Neuroscience Research Center, Physiopathology of the neuronal network responsible for the sleep-waking cycle Team, Lyon, France; 3 INSERM, U1028, Lyon Neuroscience Research Center, BioRan-Pharmaceutical and neurochemical biomarkers Team, Lyon, France; 4 CNRS, UMR5292, Lyon Neuroscience Research Center, BioRan-Pharmaceutical and neurochemical biomarkers Team, Lyon, France; 5 University Lyon 1, Lyon, France; University of Regensburg, Germany

## Abstract

Discontinuation of drug intake in cocaine abusers commonly produces a variety of adverse withdrawal symptoms among which anxiety and depression-related behavior are prevailing during the initial period of abstinence. The aim of this study was to provide further insight into the neurobiological dysregulations that might contribute to these pathological states. Rats were treated with cocaine or saline for 14 days (20 mg/kg; i.p) and anxiety-related behavior was assessed in different paradigms (elevated plus-maze (EPM), confinement to an open arm of the EPM and shock-probe burying tests) for up to 4 weeks after withdrawal. Depression-like behavior was assessed by the forced swim test and sucrose preference test. Altogether our results demonstrated that cocaine withdrawal induced persistent heightened levels of anxiety that last for at least 28 days but did not affect depression-like behavior. We then used Fos immunohistochemistry to map neuronal activation patterns in withdrawn rats confined to one open arm of an EPM, and a double labeling procedure using Fos immunohistochemistry and in situ hybridization of glutamic acid decarboxylase or vesicular glutamate transporter mRNAs to identify the phenotype of the activated neurons. Our data showed that the exacerbated anxiety observed in cocaine withdrawn rats exposed to an elevated open arm was accompanied by an altered reactivity of the dorsal part of the medial prefrontal cortex (anterior cingulate and dorsal prelimbic cortices), the paraventricular thalamic nucleus and the lateral and anterior areas of the hypothalamus. In the medial prefrontal cortex, we evidenced a negative correlation between Fos expression in its dorsal part and open arm-induced freezing in NaCl-treated rats but not in cocaine withdrawn rats. We also found that more than 65% of activated neurons were glutamatergic projection neurons. The present study provides new insights into the neuroanatomical regions and neuronal cell types that may underlie pathological anxiety during cocaine withdrawal.

## Introduction

Cocaine addiction is a chronic brain disorder characterized by compulsive drug use and high risk of relapse. Like other drugs of abuse, cocaine induces both powerful positive reinforcing effects and negative affective states that emerge when access to the drug is prevented. Indeed, discontinuation of drug intake in cocaine abusers commonly produces a variety of adverse withdrawal symptoms, among which sleep disturbances, anxiety and depression-related behavior are prevailing during the initial period of abstinence [1]. Clinical studies have also revealed a persistent hypersensitivity to stress expressed by greater emotional arousal, enhanced anxiety and endocrine reactivity [2]–[4]. These psychobiological responses to stress exacerbate with cocaine use [5] and have been suggested to increase drug abuse and relapse in addicts [6]–[9].

The mechanisms and brain regions underlying dysfunctional aspects of anxiety processing in cocaine abstinent abusers are still poorly understood. Human and animal studies on the processing of negative emotions such as anxiety have implicated several medial prefrontal cortex (mPFC) regions and a set of limbic structures which includes the amygdala, insula as well as interconnected structures such as the hypothalamus [10]–[13]. Interestingly, neuroimaging studies in abstinent cocaine dependent patients and studies in rodents have consistently pointed to persistent structural and functional alterations not only within the mesolimbic dopamine reward system [14], [15] but also in the PFC (especially in the orbitofrontal, cingulate and insular cortices) [16]–[21] and some of its connected subcortical areas (amygdala, thalamus and hippocampus) [22]–[24]. The high overlap between the brain regions affected by chronic cocaine exposure and those that are parts of the proposed anxiety circuits suggests that some of them may contribute to the impaired expression and/or regulation of anxiety during cocaine abstinence.

In order to address this issue, we first assessed in rats anxiety-like behavior in different paradigms (elevated plus-maze (EPM), confinement to an open arm of the EPM and shock-probe burying tests) for up to 4 weeks after withdrawal from chronic cocaine treatment. In addition, we concomitantly assessed depression-like behavior by the forced swim test and sucrose preference test. Indeed, although the anxiogenic and depressive-like properties of cocaine withdrawal have been documented in numerous animal studies [25]–[31], the extent to which anxiety-like behavior persists over a protracted period of withdrawal and its relation with depression-like behavior remains unclear. We then sought to examine the neurobiological alterations behind the impaired expression and regulation of anxiety levels during withdrawal. We used Fos immunohistochemistry to map neural activation patterns elicited by open arm exposure in withdrawn rats, with specific emphasis on the mPFC and its functional and cellular heterogeneity.

## Methods

### Animals

Male Sprague-Dawley rats (Charles River, France) weighing 200–225 g upon arrival were housed in groups of four and maintained in a temperature-controlled environment on a 12-hour light/dark cycle (lights on at 7:00 AM) with access to food and water *ad libitum*. Once a day for 5–7 days before the treatment, rats were handled for few minutes to reduce the stressful effects of handling. All experiments were performed between 9:30 AM and 5:00 PM.

Every effort was made to minimize animal suffering and the number of animals used. All experimental procedures were performed in accordance with the European Community Council Directives (86/609/EEC) and the French guidelines (Act 87–848 Ministère de l’Agriculture) on the care and use of laboratory animals. Authorization to conduct experiments on alive animals was obtained from the Direction départementale des services vétérinaires du Rhône (authorization number 69266242).

### Experimental Design

Rats were administered cocaine hydrochloride (COOPER, France) (20 mg/kg; i.p.) or saline (0.9%) in their home cage once a day for 14 days as previously described [28], [31], [30]. Anxiety-related behavior was assessed in different paradigms: the elevated plus maze (EPM) at 2 days of withdrawal, the confinement to one open arm of the EPM at 2 and 8 days of withdrawal and the shock probe burying test at 2, 7 and 28 days of withdrawal. Depression-like behavior was assessed in the forced swim test at 2 days of withdrawal and anhedonia was daily measured by the sucrose preference test over a withdrawal period of 4 weeks.

Independent groups of rats were used for each behavioral test and for each period of withdrawal.

### Elevated Plus-maze

A Plexiglas EPM consisting of two open (50×10 cm) and two enclosed (50×10×40 cm) arms with a black floor and arranged such as the open arms were opposite to each other was used. The maze was elevated 65 cm above the floor and located inside an isolated room with 10 lux of luminosity in the central zone.

Rats were placed in the central zone facing an open arm and their behaviors videotaped during the 5-min test period. The behavioral parameters scored were the number of entries and the time spent in the open and closed arms (one entry was counted when the rat had all 4 paws in the considered arm). Exploratory movements of the head (head-scanning) and immobility times were also monitored.

### Open Arm

For this test, one open arm of the EPM was isolated from the central zone and the closed arms of the maze by a 40 cm high wall. The open arm was divided into two equal parts, one proximal and one distal in relation to the high wall. Rats were placed in the middle of the open arm facing the open space. The behavioral parameters scored during 5 min were the time spent in the distal zone of the open arm, the number of rearing, and the time spent head-scanning and freezing (defined as complete immobility with the exception of respiratory-related movements).

### Shock-probe Burying Test

The apparatus consisted of a Plexiglas cage (25 cm wide, 41 cm long and 21 cm high) covered with 5-cm deep standard bedding material. A removable, electrified probe (7 cm long, 1.2 cm wide and 0.8 cm high; Intellibio, France) was placed through a hole 2 cm above the bedding material and was connected to a Coulbourn precision shocker (Bilaney Consultant, Germany) calibrated to deliver a shock of 0.5 mA whenever the rat touched it with a forepaw or the snout.

Rats were habituated (3–4 cage mates together) to the apparatus in the absence of the shock probe for 1 h each day for two consecutive days. On the third day, rats were tested individually with the electrified probe in place. Five min after the first shock, the current was turned off and the behaviors scored offline for 15 min following the first shock by an observer unaware of the treatment. Parameters measured were the duration of burying, the latency to return to the probe after the first shock and the total duration of immobility.

### Sucrose Preference Test

Anhedonia was assessed daily during exposure to cocaine and withdrawal by way of the sucrose preference test. Briefly, rats were housed in a single cage and were presented with two pre-weighed bottles containing either a sucrose solution or tap water. Water and sucrose consumption were measured at 10:00 AM each testing day at which time the position of the sucrose bottle (left or right) was reversed to control for side preference. Sucrose preference was calculated as the ratio of sucrose intake to total fluid intake (weights per 24 h).

### Forced Swim Test

The procedure was similar to that described previously [32]. Rats were individually placed in glass cylinders containing water (25±0.5°C) at a depth of 35 cm. Two swim sessions were conducted. A 15-min pretest was performed at 24 h of withdrawal followed by a 5-min test 24 h later. The 5-min test was recorded and analyzed by a video-tracking system (Viewpoint, Lyon, France) allowing measurements of the total duration of immobility, swimming and climbing.

### Immunohistochemistry

Two hours after the onset of the open arm test, animals were deeply anesthetized with sodium pentobarbital (50 mg/kg; i.p) and transcardially perfused with saline followed by 4% paraformaldehyde. Free-floating coronal brain sections (30 µm thick) were processed using a standard immunohistochemical procedure as previously described [33]. Briefly, a 1-in-8 series of free-floating tissue sections was taken for each rat and incubated overnight with primary antibodies in order to visualize neuronal nuclei (NeuN) (1∶8000; Chemicon International, USA), glial fibrillary acidic protein (GFAP) (1∶8000, Dakocytomation, Denmark) and Fos (1∶5000; SC-52; Santa Cruz Biotechnology, USA). The latter primary antibody reacts with rat Fos protein but not with Fos B and Δ Fos B proteins as tested by western blot (data not shown).

Rats were assigned to four groups: cocaine-treated rats and saline-treated rats exposed to the open arm; cocaine-treated rats and saline-treated rats taken in their home cage to the experiment room but not exposed to the open arm. In each experiment, sections from rats of the four groups were immunolabeled at the same time.

### Fos Immunohistochemistry Combined with GAD or vGlut1 mRNA in situ Hybridization

The antisense glutamic acid decarboxylase (GAD) probe [34] and vesicular glutamate transporter 1 (vGlut1) antisense probe [35] were obtained by in vitro transcription using a nonradioactive digoxigenin RNA labeling kit (Roche Diagnostics, Mannheim, Germany). The double labeling (Fos immunohistochemistry and mRNA in situ hybridization) was performed as previously described [36].

Images were captured using a light microscope (Axioskop 2, Carl Zeiss, Germany) equipped with a digital camera (Nikon Coolpix E995, Japan). All images were imported into Adobe Photoshop, adjusted for contrast and luminosity and assembled into plates.

### Cell Counts

**Figure 1:**
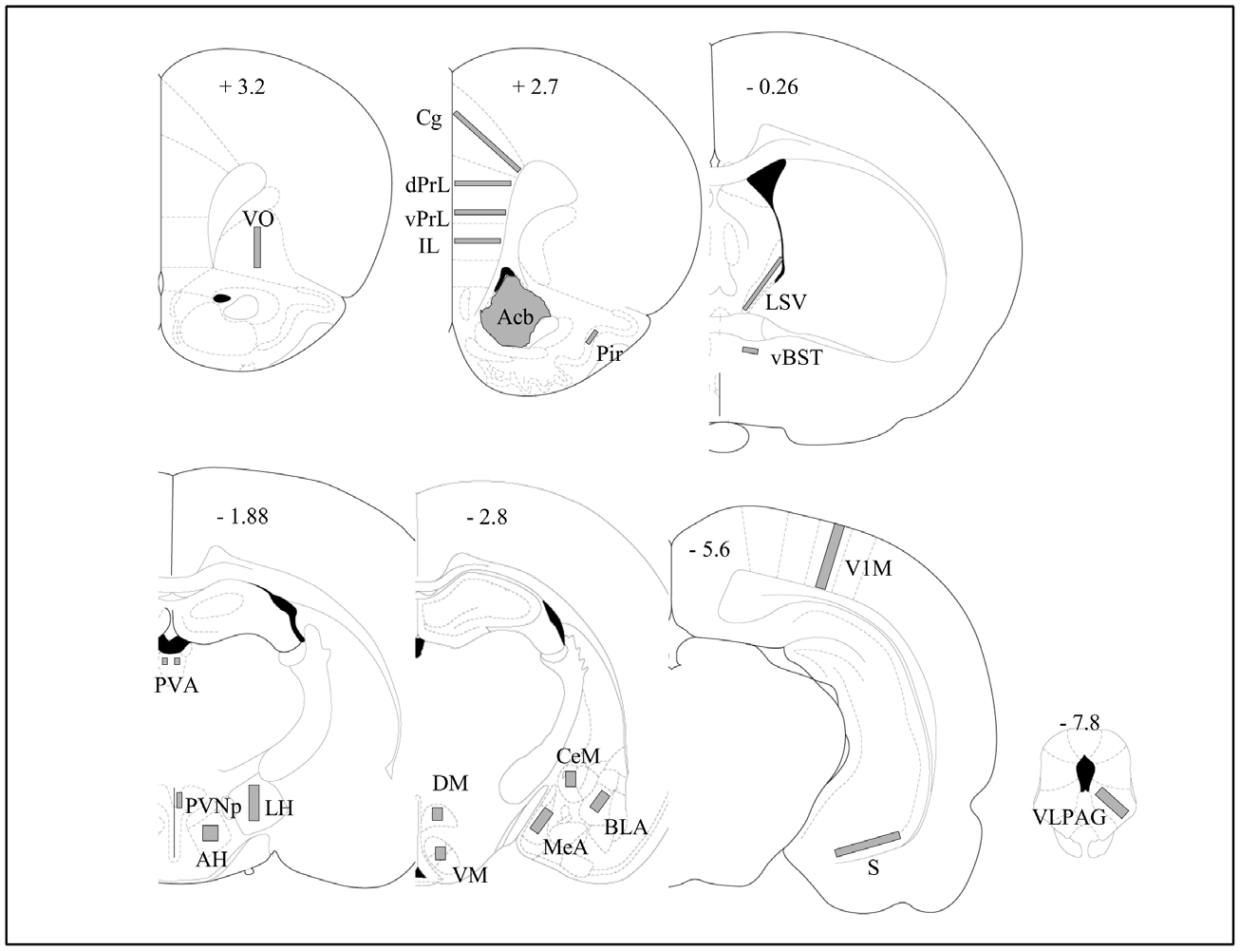
Schematic diagrams showing the areas in which Fos expression was quantified. The diagrams adapted from the atlas of Paxinos and Watson (1998) indicate the placement of grids for counting Fos-expressing cells. Bregma 3.2: VO, ventral orbital cortex; Bregma 2.7: Cg, cingulate cortex; dPrL, dorsal prelimbic cortex; vPrL, ventral prelimbic cortex; cortex; IL, infralimbic cortex; Pir, piriform cortex; Acb, accumbens nucleus; Bregma −0.26: LSV, ventral part of the lateral septal nucleus; vBNST, anteroventral part of the bed nucleus of the stria terminalis; Bregma −1.88: PVA, paraventricular thalamic nucleus; PVNp, parvocellular part of the paraventricular hypothalamic nucleus; AH, anterior hypothalamic area; LH, lateral hypothalamic area; Bregma −2.8: DM, dorsomedial hypothalamic nucleus, VM, ventromedial hypothalamic nucleus; CeM, central nucleus of the amygdala; BLA, anterior part of basolateral amygdala; MeA, medial nucleus of the amygdala; Bregma −5.6: V1M, primary visual cortex; S, subiculum; Bregma −7.8: VLPAG: ventrolateral part of the periaqueductal gray.

Bright field images of sectioned material were viewed with a Zeiss Axioskop 2 microscope equipped with an X/Y sensitive stage and a video camera (Lumenera Infinity 2) connected to a computerized image analysis system (Mercator, Explora Nova, La Rochelle, France). The planes of the analyzed sections were standardized according to the atlas of Paxinos and Watson [37]. The location of the samples used for Fos quantitative analysis is shown in Figure 1.

The quantification was carried out by first delineating the brain sections and the regions of interest (ROI) at low magnification (× 4 objective). The ROI outlines were further refined under a × 10 objective and labeled cells then counted manually by an observer blind to the experimental groups. For each rat, at least two sections per structure were quantified bilaterally. The density of labeled cells per mm^2^ was then determined and used to calculate the group means and for statistical analyses.

### Statistical Analysis

Since our data do not meet the assumptions of normality, they were analyzed by a non parametric Kruskal-Wallis test followed by a Mann-Whitney *U* test for pairwise comparisons. Correlations between Fos expression in each of the four sub-regions of the mPFC and the different behavioral scores were calculated according to the Spearman method. The sucrose consumption data were analyzed by a two-way ANOVA with one between-subject factor (treatment) and one within-subject factor (time). The level of significance was set at p<0.05. All values were expressed as mean ± SEM. Staview software was used for these statistical analyses.

## Results

### Cocaine Withdrawal Increases Anxiety-related Behavior in the Open Arm and Shock Probe Buyring Tests

**Figure 2:**
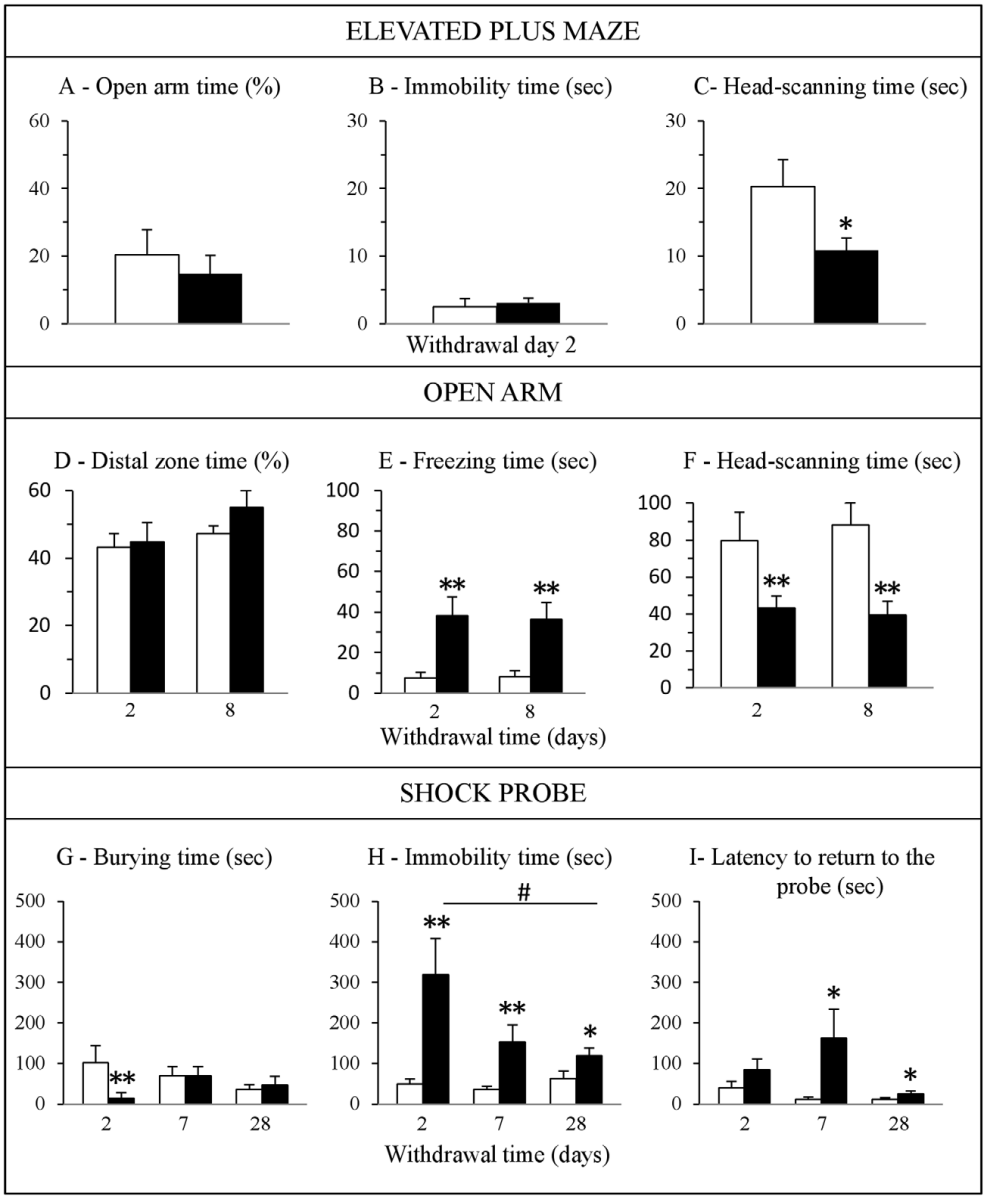
Effects of cocaine withdrawal on anxiety-related behavior. Behavior was assessed in the elevated plus-maze (A–C, n = 7–10), open arm (D–F, n = 10–14) and shock-probe burying tests (G–I, n = 10–14) in cocaine-treated rats (black bars) compared to saline-treated rats (white bars). Data are expressed as mean + SEM. *p<0.05, **p<0.01 versus saline-treated rats and ^#^ *p<*0.05 versus cocaine-treated rats at withdrawal day 2; Kruskal-Wallis test followed by a Mann-Whitney *U* test for pairwise comparisons.

Our results showed that cocaine- and saline-treated rats display no major significant differences in anxiety-related parameters in the EPM after 2 days of withdrawal. No difference was observed in the total number of entries in the open arms (2.57±0.89 vs 2.80±0.99 for saline- and cocaine-treated rats respectively), in the percentage of time spent in the open arms (Fig. 2A) or in the immobility time (Fig. 2B). The only significant difference observed was in the time spent head-scanning (Fig. 2C). No significant difference was observed in the total number of entries into open and closed arms (11.42±1.44 vs 10.9±2.03 for saline- and cocaine-treated rats respectively).

The absence of differences in anxiety-related behavior between cocaine- and saline-treated rats in the EPM led us to postulate that a more anxiogenic environment, such as confinement to the open arm [38] might be more appropriate to reveal behavioral alterations displayed by cocaine withdrawn rats. Two and 8 days after the last cocaine injection, rats exhibited longer periods of freezing during the exploration of the open arm (Fig. 2E) and less time head scanning (Fig. 2F) as well as adopting less rearing positions (Day 2: 9.75±2.19 vs 2.88±0.93 and day 8: 8.38±1.61 vs 4.14±1.68 for saline- and cocaine-treated rats respectively; p<0.05) compared to control rats. In contrast, both groups exhibited comparable percentage of time spent in the distal zone of the open arm (Fig. 2D).

Finally, cocaine-treated rats were tested during withdrawal in another well-validated model of anxiety, the shock-probe burying test, allowing the investigation of different aspects of anxiety-induced behavioral reactivity. After receiving a shock from the electrified probe, rats may exhibit a passive or avoidance behavioral response (immobility) or an active defensive burying behavior directed at the probe. At 2 days of withdrawal, periods of time spent burying the probe were less and periods of immobility longer in cocaine-treated rats compared to saline-treated rats (Fig. 2G and 2H). This increase in immobility time lasted for up to 28 days of withdrawal. Cocaine withdrawal also significantly increased the latency to return to the probe after the first shock at 7 and 28 days of withdrawal (Fig. 2I).

### Cocaine Withdrawal does not Affect Depression-related Behavior

**Figure 3:**
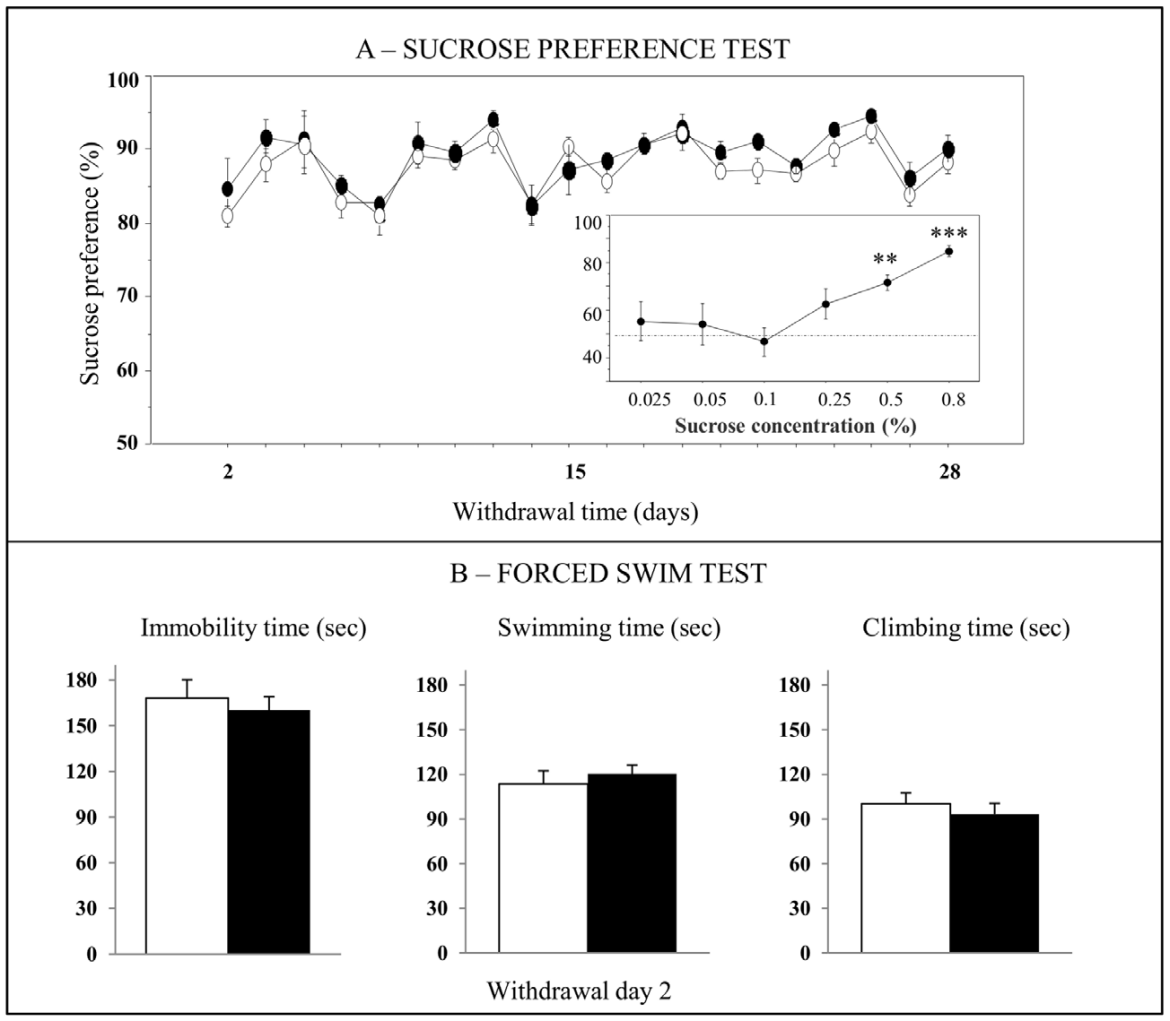
Effects of cocaine withdrawal on depression-related behavior. Behavior was assessed in the sucrose preference (A, n = 5−6) and forced swim tests (B, n = 8−7) in cocaine-treated rats (black circles or black bars) compared to saline-treated rats (white circles or white bars). (A) Evaluation of the preference for 0.5% sucrose solution during 28 days of withdrawal. The small graph on the bottom right shows the measurement of a preference for the sucrose solution at different concentrations in naive rats. The sucrose consumption data were analyzed by a two-way ANOVA with one between-subject factor (treatment) and one within-subject factor (time). (B) Measurement of different behavioral parameters in the forced swim test performed at 2 days of cocaine withdrawal. Data are expressed as mean + SEM. **p<0.01, ***p<0.001 versus saline-treated rats; Kruskal-Wallis test followed by a Mann-Whitney *U* test for pairwise comparisons.

We analyzed whether cocaine withdrawal also modified behavior in paradigms generally used to model different aspects of depression such as the sucrose preference test and the forced swim test. Naive rats were first exposed to various concentrations of sucrose (0.025, 0.05, 0.1, 0.5, and 0.8%) for 2 days in order to define the concentration allowing an increase or a decrease in sucrose consumption to be measured (Inset Fig. 3A). The sucrose concentration of 0.5% associated with a preference for sucrose of ∼70% was chosen. Rats exposed to chronic cocaine displayed no differences in total fluid intake or in sucrose preference during cocaine treatment (F_1,10_ = 0.89; ns) or throughout the following 28-day withdrawal period (F_1,8_ = 1.10; ns) (Fig. 3A). In the forced swim test, the immobility, swimming and climbing times were similar in saline- and cocaine-treated rats at 2 days of withdrawal (Fig. 3B).

### Cocaine Withdrawal Alters the Neuronal Activation Profile Elicited by Open Arm Exposure

Possible alterations in brain reactivity of cocaine withdrawn rats were explored with the expression of the immediate early gene Fos as a marker of neuronal activation. Brain structures were selected on the basis of their relevance to anxiety-related behaviors [10], [11], [39] and the density of Fos-expressing cells (Fig. 1). A few structures such as the visual and piriform cortices were chosen for controls.

**Figure 4:**
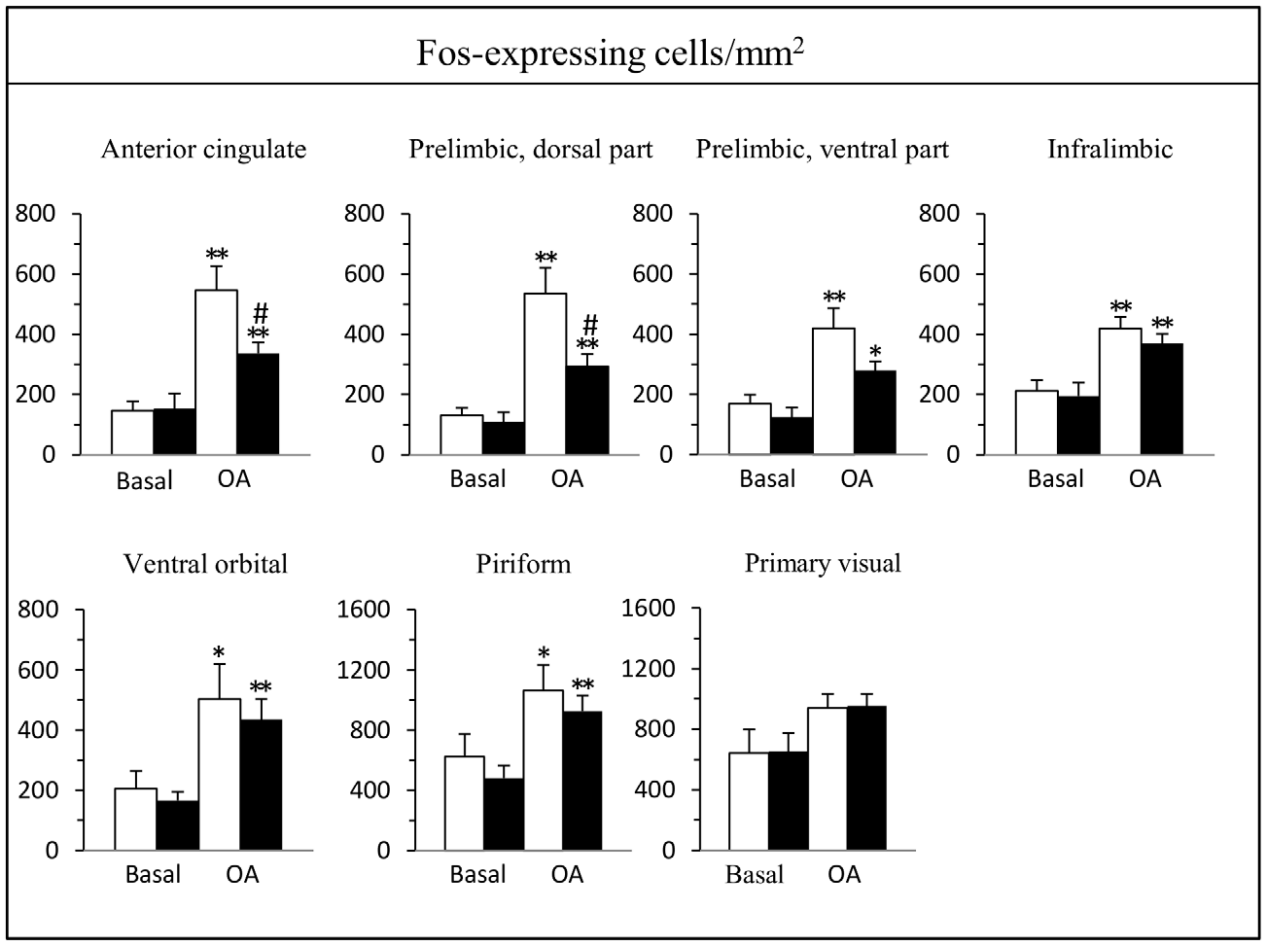
Effect of a 2-day cocaine withdrawal on Fos expression in cortical regions after open arm exposure. Cocaine-treated rats (black bars) were compared to saline-treated rats (white bars) under basal conditions (n = 5–6) and after a 5-min exposure to the open arm test (n = 7–12). Data are expressed as mean + SEM. *p<0.05, **p<0.01 versus the corresponding group of rats in basal conditions, ^#^ *p*<0.05 versus saline-treated rats placed in the open arm; Kruskal-Wallis test followed by a Mann-Whitney *U* test for pairwise comparisons.

**Figure 5:**
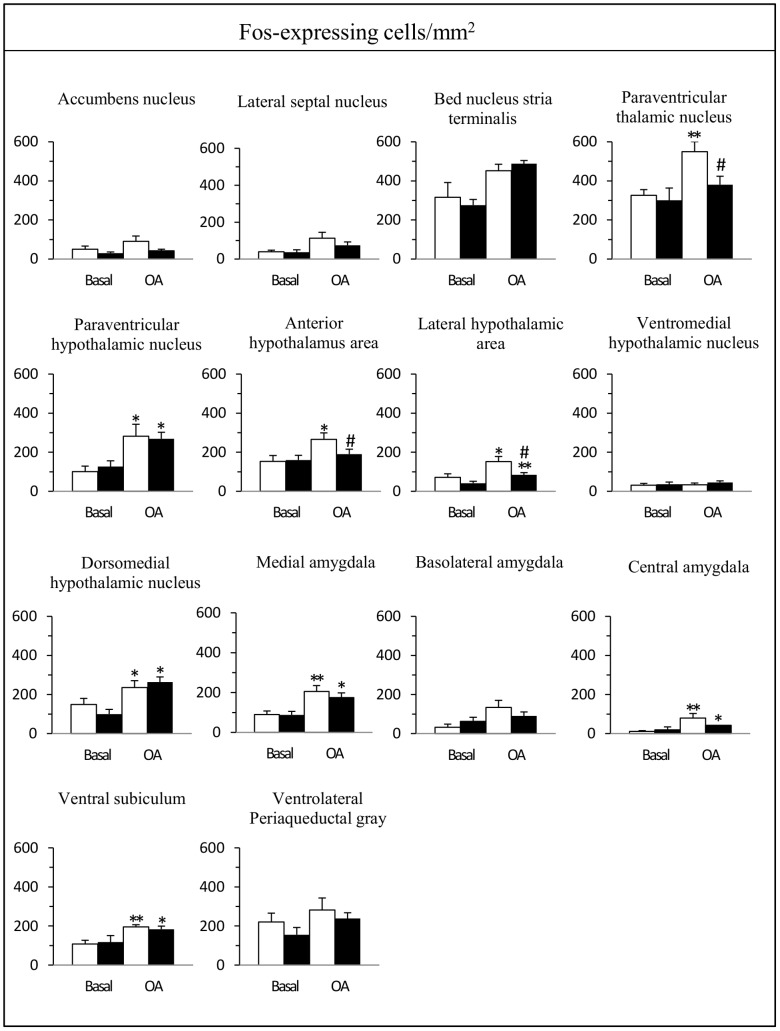
Effect of a 2-day cocaine withdrawal on Fos expression in subcortical regions after open arm exposure. Cocaine-treated rats (black bars) compared to saline-treated rats (white bars) under basal conditions (n = 5–6) and after a 5-min exposure to the open arm test (n = 7–12). Data are expressed as mean + SEM. *p<0.05, **p<0.01 versus the corresponding group of rats in basal conditions, ^#^ *p*<0.05 versus saline-treated rats placed in the open arm; Kruskal-Wallis test followed by a Mann-Whitney *U* test for pairwise comparisons.

In basal conditions, i.e. in rats not exposed to the open arm, the densities of Fos-expressing cells were low in most areas examined (<200 positive cells/mm^2^), except in the piriform and primary visual cortices and the paraventricular thalamic nucleus where the densities of Fos-expressing cells were around 600 positive cells/mm^2^. There were no significant differences between saline- and cocaine-treated rats in these regions (Figs. 4 and 5).

In cortical areas, exposure to the open arm enhanced the density of Fos-expressing cells in all subdivisions of the mPFC (anterior cingulate, dorsal and ventral prelimbic, infralimbic), ventral orbital and piriform cortices in both groups of rats whereas there was no change in the primary visual cortex (Fig. 4). Two of these 7 quantitatively evaluated areas exhibited a significantly lower Fos response in cocaine withdrawn rats, the anterior cingulate (38% reduction) and the dorsal prelimbic cortices (45% reduction) (Fig. 4). In the ventral prelimbic cortex, the Fos response in cocaine withdrawn rats was also lower (34% reduction) but did not reach statistical significance.

In subcortical areas, open arm exposure enhanced the density of Fos-expressing cells in the paraventricular thalamic nucleus, hypothalamus (paraventricular and dorsomedial nuclei, anterior and lateral areas), amygdala (medial and central nuclei) and ventral subiculum in both groups of rats (Fig. 5). It should be noted that in the hippocampal formation, the largest number of Fos-expressing cells after open arm exposure was found in the ventral subiculum whereas their number was very low in the dentate gyrus, the different subfields of Ammons’s horn and the dorsal subiculum. In the bed nucleus of the stria terminalis, Fos expressing-cells were confined to its anteroventral part, in a region that encompasses the parastriatal and dorsomedial nuclei [40]. In the periaqueductal gray, the majority of Fos-expressing cells were distributed in its ventrolateral part.

In cocaine withdrawn rats, Fos response to open arm exposure was lower only in 3 of these quantitatively evaluated areas: the paraventricular thalamic nucleus (31% reduction), the anterior (29% reduction) and lateral (46% reduction) areas of the hypothalamus (Fig. 5). In other brain regions, such as the rostral nucleus accumbens, lateral septum, bed nucleus of the stria terminalis, ventromedial hypothalamic nucleus, basolateral nucleus of the amygdala and periaqueductal gray matter, Fos expression was not significantly affected by the confinement to the open arm.

### Cocaine Withdrawal and mPFC Neuronal Activation Elicited by Open Arm Exposure

The dorsal mPFC is one of the brain regions that exhibited the highest reactivity to open arm exposure and a region of high relevance in anxiety processing and anxiety disorders. We thus went further into the cellular analysis of cocaine-induced alterations in this cortical area.

**Figure 6:**
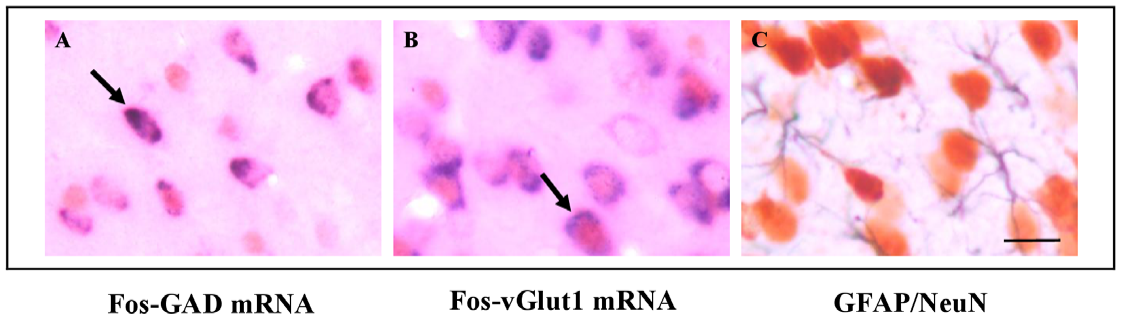
Photomicrographs showing double labeled cells in the cingulate cortex. Fos-GAD mRNA and Fos-vGlut1 mRNA expressing cells after exposure to the open arm are illustrated on photomicrographs A and B respectively. Arrows point to double labeled cells. In C is illustrated the distribution of GFAP-expressing cells (in black) and NeuN-expressing-cells (in brown). Scale bar  = 20 µm.

We first ensured that the decrease in Fos-expressing cell densities observed in cocaine withdrawn rats was not altered by cortical structural changes in terms of neuronal (NeuN-expressing cells) or astroglial cell (GFAP-expressing cells) packing densities (Fig. 6). Our results showed no significant difference in the densities of NeuN- or GFAP-expressing cells in the anterior cingulate, prelimbic or infralimbic cortices of cocaine- and saline-treated rats (Table 1). The quantification of neuronal cell densities in the mPFC allowed us to evaluate the percentage of Fos-reactive cells after open arm exposure (ratio of Fos-expressing cells on NeuN-expressing cells). This percentage did not exceed 30% in the cingulate cortex and 20% in the prelimbic and infralimbic cortices.

**Table 1:** Densities of neurons and astroglial cells and proportion of Fos-positive cells expressing GAD mRNA or vGlut1 mRNA in the mPFC.

	Infralimbic cortex		Prelimbic cortex		Anterior Cingulate cortex	
	Saline	Cocaine	Saline	Cocaine	Saline	Cocaine
**NeuN**	2275.11±85.05	2376.12±120.38	2089.38±96.64	2218.24±72.7	1546.26±46.61	1927.91±50.18
**GFAP**	722.19±77.75	646.03±62.91	607.13±77.89	668.25±134.46	496.23±60.23	509.4±32.74
**% Fos-GAD**	35.46±4.42	28.32±2.59	31.05±3.20	25.22±2.81	35.26±8.58	36.52±6.51
**% Fos-vGlut1**	65.56±6.14	66.15±3.66	71.92±5.23	78.69±3.41	75.79±4.23	79.51±5.96

Quantification was performed in infralimbic, prelimbic and anterior cingulate cortices of cocaine- and saline-treated rats after open arm exposure. Data are expressed as mean ± SEM of three rats from each group and represent the density of labeled cells/mm^2^ or the percentage of Fos-expressing cells containing the mRNAs coding for vGlut1 or GAD.

We then sought to determine the phenotype of the cortical Fos-activated neurons. Double labeling of Fos-labeled cells with vGlut1 mRNA or GAD mRNA (Fig. 6) indicated that Fos activation occurred in glutamatergic (more than 65% of Fos-activated neurons) as well as GABAergic neurons (about 35% of Fos-activated neurons), percentages that were maintained between groups (Table 1).

Finally, we examined if there was a correlation between Fos expression in the different subdivisions of the mPFC and the behavioral data obtained in the open arm. Interestingly, we found a significant and selective negative correlation between Fos expression in the cingulate (r = −0.89, p<0.01), dorsal prelimbic (r = −0.93, p<0.05) cortices and the time spent freezing in NaCl-treated rats. This negative correlation was disrupted in cocaine withdrawn rats.

## Discussion

Two major findings have emerged from the present study. First, we showed that cocaine withdrawal had a sustained effect on anxiety-related behavior, particularly that in response to highly challenging situations. In contrast, the same treatment did not affect depression-like behavior suggesting that in our experimental conditions, cocaine had a more profound effect on the mechanisms involved in anxiety regulation than those involved in mood regulation. Second, our anatomical data provide new insight into the neural substrates that may underlie pathological anxiety during cocaine withdrawal. Indeed, we showed that exacerbated anxiety observed in cocaine withdrawn rats placed in an anxiogenic environment was accompanied by dysfunctions within a restricted set of neuroanatomical regions including the dorsal mPFC and anatomically-related subcortical regions such as the paraventricular thalamic nucleus and specific regions of the hypothalamus.

Our behavioral data increased our knowledge on the affective states induced by withdrawal from chronic cocaine exposure in rodents. While several studies have already reported a similar elevation of anxiety-related behavior in rodents exposed to the EPM [28], [30], [31] here we have shown that this effect is more readily observed and more sustained in rats having to cope with a higher anxiogenic environment [38] or an aversive stimulus [41]. This heightened anxiety state was evidenced by long bouts of freezing in the open arm that persisted for at least 8 days or immobility in the shock probe burying tests that persisted for at least 28 days of withdrawal. These passive behaviors cannot be interpreted as an impaired motor activity since cocaine withdrawn rats exhibited a comparable total number of entries into the distal zone of the open arm, or total open and closed arms in the EPM.

These behavioral findings suggest that cocaine-induced neuroadaptations contribute to long-lasting impairment in the ability to cope with anxiogenic or stressful situations following drug exposure and withdrawal. They support previous observations indicating that persistent heightened levels of anxiety are expressed after protracted withdrawal periods when rats are placed in highly challenging situations such as that during the defensive burying test procedure [25], [26], [41] or after exposure to contextual cues previously paired with cocaine [42], [43]. Our data are also relevant with regards to clinical studies highlighting a persistent hypersensitivity to stress during abstinence in cocaine abusers [2]–[4].

In contrast, we found no alterations in behavior related to depression. We observed no evidence of helplessness or anhedonic states during withdrawal, two different aspects of depression that were respectively assessed by the forced swim test and the sucrose preference test. Previous data addressing this issue have yielded equivocal results, with some reporting no change [44] and others alterations in behavior in the forced swim test during early phase of cocaine withdrawal [29], [45], [46]. Regarding anhedonic-like symptoms during cocaine withdrawal, they have been only reported in experiments using the intracranial self-stimulation procedure as a measure of brain reward system function [47], [48]. Thus, the discrepancies observed between the different reports and our data might be due to procedural differences including the amount and pattern of cocaine administration (active vs passive, daily vs binge administrations) and the testing procedure. Although we observed no effect of chronic cocaine treatment on depression-related behavior in our experimental conditions, we may hypothesize that cocaine-induced disruption in anxiety regulation compromises stress coping and increases vulnerability to depression mediated by stressful life events. Further investigations are needed to address this specific issue.

To the best of our knowledge, no previous anatomical study has directly addressed the identification of the neural circuit dysfunctions underpinning the impaired expression or regulation of anxiety observed during cocaine withdrawal. Previous studies aiming at characterizing the neurobiological substrates of cocaine-induced anxiety have focused on the action of different receptors such as corticotropin-releasing factor [31], [41], beta-adrenergic [26], [30], cannabinoid [49] and delta opioid [29] receptors. Here, using Fos immunohistochemistry, we showed that open arm exposure activates a large number of cortico-limbic areas also stimulated by different innate anxiety paradigms [11], [39], [50]. In addition, we demonstrated that the enhanced anxiety observed in cocaine withdrawn rats was associated with altered neuronal processing in specific key areas of the brain. Indeed, whereas regions such as the infralimbic cortex, bed nucleus of the stria terminalis, dorsomedial hypothalamic nucleus, central and medial nuclei of the amygdala, ventral subiculum, or ventrolateral periaqueductal gray did not show differential activation in cocaine withdrawn rats compared to NaCl-treated rats, a small set of interconnected regions including the cingulate and dorsal prelimbic cortices, paraventricular thalamic nucleus, lateral and anterior hypothalamic areas exhibited a striking significantly lower number of Fos-expressing neurons in cocaine-withdrawn rats.

This specific neuroanatomical effect in cocaine withdrawn rats is a novel finding of interest in many respects. Our results are in line with observations made in two other rodent models of pathological anxiety, high anxiety BALB/c mice [51] or rodents selectively bred for high anxiety-related behavior [50], [52]. In these rodents, a blunted Fos response to anxiogenic situations was revealed in the cingulate cortex, but not in the prelimbic and infralimbic cortices, when compared to their low anxiety counterparts (respectively C57BL/6 mice and rodents selected for their low-anxiety related behavior). Moreover, these studies, like our own, pointed to altered Fos responses in subcortical areas such as the paraventricular thalamic nucleus, anterior and lateral hypothalamic areas. Interestingly, the paraventricular thalamic nucleus and the lateral hypothalamus areas are interconnected to the mPFC [53]–[56] and have been involved in different aspects of anxiety [57], [58]. Our data thus suggest a possible link between these dysfunctions in corticolimbic network and exacerbated anxiety induced by cocaine.

The differential effect of cocaine withdrawal on the reactivity of the dorsal and ventral subdivisions of the mPFC is relevant in regard to the complex organization of this structure. Anatomical and functional studies have demonstrated a dorsoventral gradient along the mPFC, with the dorsal part (that includes the anterior cingulate and dorsal prelimbic cortices) primarily interconnected to regions that reportedly affect cognition, and the ventral part (that includes ventral prelimbic and infralimbic cortices) connected to autonomic/visceral related regions [53], [56], [59]. Consistent with this, multiple lines of evidence demonstrated that the ventral mPFC plays an important role in the generation of anxiety, based on the fact that focal lesions or local inactivation of this area have an anxiolytic effect in rats [60], [61] and reduce the autonomic [62] and hormonal [63] responses to acute emotional stress. The role of the dorsal mPFC in innate anxiety is more controversial since some studies reported anxiolytic effects [60], [64] while others reported anxiogenic effects [65], [66] after its inactivation. This may be due to its modulatory influence on emotional behavior and the fact that its involvement may vary according to the anxiogenic situations the rodents have to face with [67]. The negative correlation we found between Fos-expression in the dorsal prefontal cortex and the time NaCl-treated rats spent freezing in the open arm in rats as well as its disruption during cocaine withdrawal questions the relationship between cortical activity and anxiety-related behavior. In view of these results, we may hypothesize that the high anxiety levels observed in cocaine withdrawn rats is linked to a poor recruitment of dorsal mPFC neurons leading to a decreased control over emotional and cognitive aspects of anxiety-related behavior.

Our animal model also allowed us to go further into the cellular analysis of cocaine-induced alterations in the mPFC without the confounding obstacles commonly encountered in human studies such as polydrug abuse, premorbid psychiatric conditions and variable drug history. Since previous studies have reported the remodeling of cortical neuron dendrites [14], [20], significant modifications in glial and neuronal cell number [68], [69] and microischemia [70] in the forebrain after chronic cocaine treatment, we first ensured that the decrease in Fos-expressing cell densities observed in the cocaine withdrawn rats was not confounded by structural changes in the cortex that could have led to modifications in neuronal cell densities. We next demonstrated that open arm exposure activated a majority of cortical glutamatergic pyramidal neurons (65 to 80% of activated neurons) whereas the remaining activated cells were GABAergic interneurons. This ratio, which concords with the estimated proportion of these two populations of neurons in various cortical areas [71], was not affected in cocaine withdrawn rats. We conclude therefore that cocaine has no preferential action on the reactivity of glutamatergic or GABAergic cortical neurons involved in anxiety processing. However, how these subsets of cingulate and dorsal prelimbic glutamatergic outputs act to shape the high levels of anxiety observed in cocaine-treated rats is an important question to address in the future.

Although Fos is considered as a generic marker of acute neuronal activation and/or changes in cellular signaling [72] its expression might be modified by previous pharmacological treatment. It has been shown that chronic cocaine exposure induced the accumulation of ΔFosB, a transcription factor that persists few days after withdrawal [73]–[76] and causes long-lasting cellular and molecular changes [77] including negatively regulation of Fos expression [78]. Thus, if ΔFosB is expressed in neuronal circuits involved in the expression or regulation of anxiety, it could also account for the attenuated Fos response observed in cocaine withdrawn rats and for dysregulations of their neuronal function [77], but not necessarily for a hyporesponsiveness of these circuits. In addition, although our Fos anatomical data are determinant for identifying the brain regions that may contribute to enhanced anxiety in cocaine withdrawn rats they remain correlative. Clearly, further investigations are needed to determine whether the altered dorsal mPFC reactivity, for example, has a causal role in the exacerbated anxiety behavior observed in cocaine withdrawn rats. Experiments allowing transient activation or inactivation of this sparsely distributed neural system would be suitable to address this issue.

In conclusion, the present study provides new insights into the neuroanatomical regions and neuronal cell types that may underlie cocaine-induced disruption in ability to cope with highly anxiogenic situations. Our observations reinforcing the potential importance of the PFC in the negative sequelae of chronic cocaine exposure is in consonance with previous studies reporting neuroadaptations in cortico-striatal-limbic circuits that underlie behavioral and cognitive aspects of cocaine dependence [14], [19]. This animal model offers not only the opportunity to decipher the underlying pathophysiological mechanisms of cocaine-induced anxiety, but also to develop new pharmacological tools for its treatment.
